# Recombinant VP1, an Akt Inhibitor, Suppresses Progression of Hepatocellular Carcinoma by Inducing Apoptosis and Modulation of CCL2 Production

**DOI:** 10.1371/journal.pone.0023317

**Published:** 2011-08-03

**Authors:** Tai-An Chen, Jui-Ling Wang, Shao-Wen Hung, Chiao-Li Chu, Yung-Chih Cheng, Shu-Mei Liang

**Affiliations:** Agricultural Biotechnology Research Center, Academia Sinica, Taipei, Taiwan; University of Hong Kong, Hong Kong

## Abstract

**Background:**

The application of viral elements in tumor therapy is one facet of cancer research. Recombinant capsid protein VP1 (rVP1) of foot-and-mouth disease virus has previously been demonstrated to induce apoptosis in cancer cell lines. Here, we aim to further investigate its apoptotic mechanism and possible anti-metastatic effect in murine models of hepatocellular carcinoma (HCC), one of the most common human cancers worldwide.

**Methodology/Principal Findings:**

Treatment with rVP1 inhibited cell proliferation in two murine HCC cell lines, BNL and Hepa1-6, with IC_50_ values in the range of 0.1–0.2 µM. rVP1 also induced apoptosis in these cells, which was mediated by Akt deactivation and dissociation of Ku70-Bax, and resulted in conformational changes and mitochondrial translocation of Bax, leading to the activation of caspases-9, -3 and -7. Treatment with 0.025 µM rVP1, which did not affect the viability of normal hepatocytes, suppressed cell migration and invasion via attenuating CCL2 production. The production of CCL2 was modulated by Akt-dependent NF-κB activation that was decreased after rVP1 treatment. The in vivo antitumor effects of rVP1 were assessed in both subcutaneous and orthotopic mouse models of HCC in immune-competent BALB/c mice. Intratumoral delivery of rVP1 inhibited subcutaneous tumor growth as a result of increased apoptosis. Intravenous administration of rVP1 in an orthotopic HCC model suppressed tumor growth, inhibited intra-hepatic metastasis, and prolonged survival. Furthermore, a decrease in the serum level of CCL2 was observed in rVP1-treated mice.

**Conclusions/Significance:**

The data presented herein suggest that, via inhibiting Akt phosphorylation, rVP1 suppresses the growth, migration, and invasion of murine HCC cells by inducing apoptosis and attenuating CCL2 production both *in vitro* and *in vivo*. Recombinant protein VP1 thus has the potential to be developed as a new therapeutic agent for HCC.

## Introduction

Hepatocellular carcinoma (HCC) is one of the most common cancers worldwide [1]. Although the etiology of HCC has been largely explained, HCC is still a focus of cancer research due to its poor prognosis and high rates of recurrence resulting from local invasion and intra-hepatic metastasis [2], [3]. Since HCC is highly resistant to conventional cytotoxic chemotherapy, most recent investigations have focused on molecular targeted therapies. The Ras/Raf/Mek/Erk and PI3K/Akt/mTOR signaling pathways are important in hepatocarcinogenesis, and several inhibitors targeting those pathways are currently under clinical development [4]–[7]. Despite these advances, the continued investigation of new therapeutic modalities for HCC is warranted due to the shortage of effective systemic therapy for advanced cases and the highly unfavorable prognosis of the disease.

Bacterial and viral elements that promote cancer cell death have long been described in the literature. Azurin, a periplasmic protein secreted by *Pseudomonas aeruginosa*, inhibits the growth of various cancer cells and induces apoptosis upon cell entry [8]. Apoptin and E4orf4, viral proteins derived from chicken anemia virus and human adenovirus, respectively, have been shown to induce cell apoptosis and tumor regression in various pre-clinical animal models [9]–[11]. Previous studies thus indicate that bacterial or viral proteins can be exploited for treatment of cancers.

Foot-and-mouth disease virus (FMDV), an etiological agent of FMD, is composed of 60 copies of each of four capsid proteins termed VP1 to VP4. These proteins form a closed capsid surrounding a long single strand of RNA [12]. We previously purified a water-soluble, recombinant VP1 protein of FMDV (rVP1) and found that it induced apoptosis of BHK-21 cells through binding to integrins [13]. The apoptosis-related events shown in rVP1-treated BHK-21 cells included DNA fragmentation, Akt deactivation, and enhancement of several pro-apoptotic responses such as dephosphorylation of GSK-3β and cleavage of pro-caspases-9, -7 and -3. In addition, treatment with rVP1 caused apoptosis in three cancer cell lines: the breast carcinoma cell line MCF-7, the androgen-independent prostate cancer cell line PC-3, and the androgen-dependent prostate cancer cell line 22Rv1 [13]. However, the molecular mechanism underlying apoptosis in cancer cells induced by rVP1 remains unclear.

The Bcl-2 (B-cell lymphoma 2) family of proteins regulates cell apoptosis by controlling the integrity of mitochondria. Bax (Bcl-2-associated X protein), a crucial member of pro-apoptotic Bcl-2 family proteins, is largely retained in the cytosol by some suppressor proteins in a quiescent state while survival signal dominates. Ku70, the 70-kDa subunit of the Ku protein complex composed of Ku70 and Ku80, is a Bax suppressor protein [14]. It has been demonstrated that the interaction between Ku70 and Bax is maintained by Akt activation [15]. Upon apoptotic stress, the level of Ku70 is decreased, allowing the dissociation of Bax from Ku70 [14], [15]. In addition, Bax undergoes a conformational change (activation) followed by mitochondrial translocation and insertion into the outer mitochondrial membrane, leading to the initiation of a downstream caspase cascade and subsequent apoptosis [16].

Emerging evidence suggests that the CC chemokine CCL2/MCP-1 (monocyte chemotactic protein-1) plays pleiotropic roles in cancer development. Hepatic myofibroblasts have been shown to secret CCL2 to promote migration and invasion of hepatoma cells [17]. Moreover, a higher mRNA level of CCL2 is found in human HCC [18]. Several other types of cancer cells, including prostate cancer, breast cancer, and myeloma cells, have also been demonstrated to express CCL2 and its receptor CCR2 [19]–[21], both of which are correlated with prostate cancer development [19], [22]. In patients with breast or ovarian cancer, high serum levels of CCL2 positively correlated with tumor development [23], [24]. On the other hand, CCL2 blockade inhibited tumor growth and metastasis in lung cancer mouse models [25]. Mounting evidence also indicates that CCL2 is a key player in the development of bone metastases [26], although its function in bone lesion formation has not been fully elucidated. In addition, CCL2 has also been associated with cell protection [27], [28].

In this study, we aimed to investigate the pro-apoptotic and anti-metastatic/anti-invasive effects of rVP1 on HCC *in vitro* and *in vivo*. We propose that the apoptosis induced by rVP1 occurs through a mechanism that involves dissociation of Bax from Ku70. Furthermore, rVP1 inhibited metastasis/invasion via attenuating CCL2 production. *In vivo* experiments, using both subcutaneous and orthotopic mouse models of HCC, revealed that rVP1 suppressed tumor growth, inhibited intra-hepatic metastasis, and showed survival benefit.

## Materials and Methods

### Cell line and culture conditions

Murine hepatocellular carcinoma cell lines BNL 1 ME A.7R.1 (BNL) and Hepa1-6 were kindly provided by Dr. Mi-Hua Tao, Institute of Biomedical Sciences, Academia Sinica (Taipei, Taiwan). The BNL and Hepa1-6 cells were maintained in Dulbecco's modified Eagle's medium (DMEM; Gibco, Gaithersburg, MD) supplemented with 10% heat-inactivated fetal bovine serum (FBS; Gibco), 2 mM L-glutamine, 100 U/ml penicillin and 100 µg/ml streptomycin in a humidified incubator at 37°C under 5% CO_2_. The AML 12 (alpha mouse liver 12) cell line derived from normal murine hepatocytes was purchased from the Bioresource Collection and Research Center (Hsinchu, Taiwan) and maintained in a mixture of DMEM and Ham's F12 medium supplemented with 0.005 mg/ml insulin, 0.005 mg/ml transferrin, 5 ng/ml selenium (Gibco), 40 ng/ml dexamethasone (Sigma, St. Louis, MO), and 10% FBS.

### Purification of recombinant VP1 proteins

Purification of recombinant VP1 proteins was carried out according to procedures published previously [13], [29]–[31]. In brief, the VP1 gene with a T7 and a His tag at the N- and C-terminus, respectively, was ligated between the BamHI and XhoI sites of pET24a(+) (Novagen, Madison, WI), and then expressed in BL21 (DE3) *Escherichia coli* (Stratagene, La Jolla, CA). The recombinant VP1 was isolated by breaking up the bacterial cells with a Microfluidizer in TEN buffer (50 mM Tris-HCl, pH 8.0, 1 mM EDTA, 0.1 M NaCl). The resultant cell lysate was centrifuged and the pellet was washed three times with 0.5% deoxycholate in TEN buffer. After rinsing with TEN buffer, the pellet was resuspended in freshly prepared binding buffer (20 mM Tris–HCl, pH 8, 0.5 M NaCl, 8 M urea). The solution was then applied to a metal-chelating affinity column and the fractions containing rVP1 protein were collected. SDS was then added to the protein solution to a final concentration of 1%. The protein solution was subsequently applied to a Superdex 200 column (Amersham, UK) equilibrated with a buffer solution containing 25 mM Tris-HCl, pH 8.0, 1 mM EDTA, 0.1 M NaCl, and 0.05% SDS. Fractions containing rVP1 protein were identified by SDS-PAGE and pooled. The protein was concentrated and dialyzed against PBS before use.

### Cell growth inhibition assay

Cells maintained in medium with 10% FBS were seeded in 96-well plates at a density of 2×10^4^ cells/well overnight. The wells were washed with PBS buffer (Gibco) prior to the addition of rVP1 at various concentrations, diluted with serum-free medium, and incubated for 16 h. An MTT assay was then used to evaluate the cell viability, and the concentration of rVP1 required to inhibit cell growth by 50% (IC_50_) was determined by interpolation from the concentration-response curve.

### Flow cytometric analysis of apoptotic cells

For evaluation of annexin V activity, cells were treated with 1 µM rVP1 for 16 h and then detached for labeling. Cells were collected by centrifugation, resuspended in binding buffer, and incubated with annexin V-FITC and propidium iodide (Annexin V-FITC apoptosis detection kit, Biovision, Mountain View, CA) for 5 minutes in the dark before flow cytometric analysis on a FACSCalibur system (BD, Franklin Lakes, NJ).

### Mice and subcutaneous allograft model of HCC

BALB/c mice were purchased from the National Laboratory Animal Center (Taiwan). All animal experiments were approved by the Institutional Animal Care and Utilization Committee of Academia Sinica, Taiwan (approval ID: MMiIBALS 2006069). BNL cells (5×10^6^ cells per mouse) were suspended in 100 µl of serum-free DMEM and injected subcutaneously into mice, aged 6 to 8 weeks. When tumors were detected, tumor volume was measured using the formula: 1/2×the largest diameter×(the smallest diameter)^2^, as reported in previous cancer studies [32]–[34]. Mice with similar tumor volumes (about 250 mm^3^) were randomly sorted into groups for intratumoral injection of rVP1. The animals were administered rVP1 (25 to 100 mg/kg) three times a week for a total of 9 treatments. Measurement of tumor volume was periodically performed with calipers. At the end of rVP1 treatment, two mice in each group were sacrificed for western blot and immunohistochemical analyses.

### Orthotopic allograft HCC model

An orthotopic HCC model was established by intra-hepatic implantation of BNL cells as described previously [35]. Briefly, BALB/c mice were anaesthetized with isoflurane, and a small transverse incision was made in the left abdomen. One hundred thousand BNL cells suspended in 20 µl PBS containing 50% Matrigel (BD Biosciences, Bedford, MA) were slowly injected with a 30-gauge needle into the left liver lobe which was exposed through the incision. After injection, the incision was closed by suture with absorbable material. One day after the surgery, mice received the first dose of rVP1 or PBS via tail vein. Mice were subsequently injected three times a week for 3 weeks. Mice sacrificed after five rVP1 treatments were used for pathological analysis.

### Detection of cytokine/chemokine production

Serum levels of CCL2/MCP-1, IL-6, IL-10, IL-12p70, IFN-γ, and TNF in mice bearing subcutaneous BNL tumors were measured by cytometric bead array assay (Mouse Inflammation Kit; BD Biosciences). Production of CCL2, IL-6 and TNF-α by BNL cells was measured by ELISA. Briefly, BNL cells were seeded in a 24-well plate and cultured overnight before addition of rVP1 or NF-κB activation inhibitor, 6-amino-4-(4-phenoxyphenylethylamino)quinazoline (InSolution™, Calbiochem, San Diego, CA). After incubation for 48 h, the levels of cytokines present in culture supernatants of BNL cells treated with or without rVP1 were quantified by commercial ELISA kits (eBioscience, San Diego, CA). The number of viable cells in individual wells was determined by MTT assay and used to normalize the ELISA data.

### Immunohistochemistry

Apoptotic cells in frozen sections of subcutaneous allografts were detected with a TumorTACS In Situ Apoptosis Detection Kit (R&D Systems, Minneapolis, MN) following the manufacturer's instructions. Five fields per slide were examined at 400× magnification, and the percentage of positively stained cells was calculated using the AxioVision v4.6 image processing software (Zeiss, Germany). The paraffin-embedded orthotopic tumors were processed according to standard immunohistochemistry protocols. Antigen retrieval was performed with Target Retrieval Solution (S1700, DAKO), followed by quenching of endogenous peroxidase activity. After blocking, sections were incubated with anti-Ki-67 or anti-Ku70 antibody (Biocare Medical, Concord, CA) at 4°C overnight in a humid chamber. An ABC staining system (Santa Cruz Biotechnology, St. Louis, MO) was used to detect the reaction products. *In situ* detection of apoptotic cells was carried out with the TumorTACS kit as described above.

### Western blot analysis

Total proteins were extracted using protein extraction reagents (Pierce, Rockford, IL) from cell line lysates or homogenized tumor specimens removed 24 h after the last rVP1 injection. The concentration of protein extract was determined using the BCA protein assay kit (Pierce). The protein extracts were resolved by 4–12% SDS-PAGE (30–50 µg of protein/lane), transferred onto a PVDF membrane (Millipore, Bedford, MA), and probed with specific antibodies, including anti-pAkt, anti-Akt, anti-pro-caspases, anti-cleaved-caspases, and anti-pIKK (Cell Signaling, Beverly, MA); anti-IKKα (Upstate); anti-Ku70 (H-308, Santa Cruz Biotechnology); anti-conformationally active BAX (clone 6A7, BD Pharmingen, San Diego, CA); as well as anti-actin and anti-GAPDH (Chemicon, Temecula, CA). The blots were developed using chemiluminescent substrates (Pierce).

### Immunoprecipitation

For detection of Ku70-Bax interactions in BNL and Hepa1-6 cells, cells treated with or without rVP1 were harvested in protein extraction reagents (Pierce), and 0.2–1 mg of cell lysates were immunoprecipitated with 4–10 µg of anti-Bax antibody (Sigma). The immunocomplexes were captured using an immunoprecipitation matrix (ExactaCruz C, Santa Cruz Biotechnology) following the manufacturer's protocol. Mouse IgG (Zymed, San Francisco, CA) was used as a negative control. After washing with PBS five times, western blot analysis of pre-immunoprecipitated (Input) and immunoprecipitated (IP) samples was performed with an anti-Ku70 antibody.

### Luciferase activity assay

BNL cells (1×10^4^) were seeded into each well of a 96-well plate 24 h before transfection. Cells were then transiently transfected with 0.06 µg of NF-κB-luciferase reporter plasmid, 0.02 µg of EGFP plasmid, and 0.02 µg of wild type/dominant active Akt or vector control plasmid (Upstate) in serum-free medium using the Lipofectamine™ 2000 reagent (Invitrogen). After incubation for 6 h, the medium was replaced with fresh DMEM containing 10% FBS. On the following day, the cells were treated with 1 µM rVP1 in 0.5% (v/v) FBS/DMEM for 6 h and the luciferase activity was determined using the luciferase assay system with lysis buffer from Promega. The results are expressed as relative NF-κB activity compared with controls after normalizing for EGFP values.

### Boyden chamber migration/invasion assay

Membranes of insets (8 µm pore size, Corning, Corning, NY) were coated with fibronection (20 µg/ml, Millipore) or Matrigel (500 µg/ml, BD Biosciences) for measurement of cell migration or invasion, respectively. Cells (1×10^4^) were seeded in the inserts in serum-free DMEM, and media supplemented with 10% FBS were placed in the lower chamber. One hour later, rVP1 with or without recombinant mouse CCL2 proteins (Peprotech, Rocky Hill, NJ) were added into inserts followed by incubation for 24 h. Inserts were then removed from the chambers and submerged in methanol to fix cells. Non-migrated cells on the upper side of the membrane were removed by gently scraping the top side of the insert with a cotton swab. Cells that migrated to the other side of the membrane were stained with Liu's stain (Muto Pure Chemicals, Tokyo, Japan). The number of migrated/invaded cells was calculated under a microscope at 100× magnification.

### Statistical analysis

Testing for statistically significant differences between two groups of data was done using a 2-tailed Student's *t* test. The log-rank test was used to compare the survival curves of mice with orthotopic HCC. ELISA data were compared using ANOVA with Dunnett's post test. All data analyses were performed with GraphPad Prism 5.0 for Microsoft Windows (GraphPad Software, La Jolla, CA). *P* values less than 0.05 were considered statistically significant. ^*^ *P*<0.05; ^**^ *P*<0.01; ^***^ *P*<0.001.

## Results

### rVP1 inhibits cell growth and induces apoptosis in HCC cells

**Figure 1:**
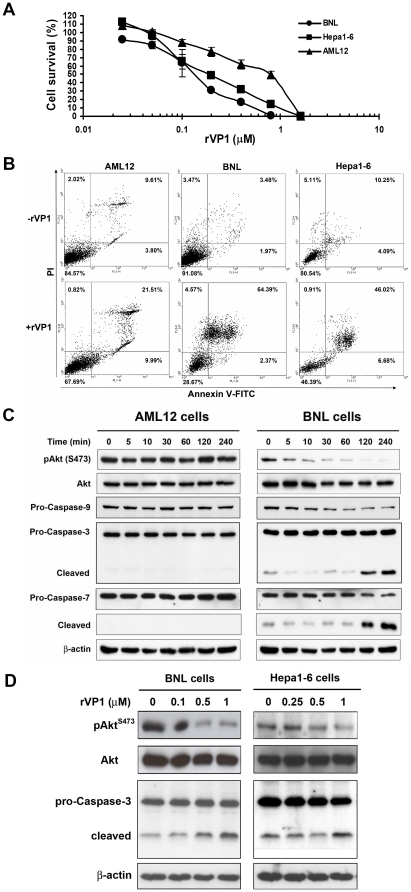
Treatment with rVP1 inhibits growth and induces apoptosis in HCC cell lines. (A) Cells were treated with various concentrations of rVP1 in serum-free medium for 16 h and assayed for viability. All assays were performed in triplicate, and data shown are representative of three independent experiments. (B) Flow cytometric detection of apoptosis by annexin V and propidium iodide (PI). Cells were treated with or without 1 µM rVP1 in serum-free medium for 16 h. (C) Western blots of phospho-Akt (pAkt) and cleavage of pro-caspase-9, -3 and -7. BNL and AML12 cells were treated with 1 µM rVP1 for the times indicated. (D) Dose-dependent effect of rVP1 on pAkt and cleavage of pro-caspase-3 in BNL and Hepa1-6 cells. Cells were treated with indicated concentrations of rVP1 for 1 and 4 h for pAkt and caspase 3 detection, respectively.

Previously, we reported that rVP1 induces apoptosis in breast and prostate cancer cell lines [13]. To investigate whether rVP1 holds any potential for treatment of HCC, we first examined its effect on cell growth and induction of apoptosis in HCC cells. As shown in Figure 1A, treatment with rVP1 inhibited cell proliferation in two HCC cell lines, BNL and Hepa1-6, and a normal murine hepatocyte cell line, AML12, in a concentration-dependent manner. The IC_50_ values of rVP1 for BNL, Hepa1-6 and AML12 cells were 0.14, 0.19 and 0.79 µM, respectively. The ability of rVP1 to induce apoptosis was analyzed by annexin V-FITC/propidium iodide (PI) staining and flow cytometry. As shown in Figure 1B, treatment with rVP1 resulted in HCC cell death via apoptosis. Activation of the PI3K/Akt pathway is known to cause cell proliferation, whereas deactivation of Akt causes apoptosis. Therefore, we next examined whether rVP1 treatment could modulate the level of pAkt and its downstream apoptosis-associated caspase activation. When murine HCC cells were treated with rVP1, Akt phosphorylation was inhibited in a time- and dose-dependent manner (Figures 1C and D). By contrast, such an effect was not observed in AML12 cells. Western blot analysis of mitochondria-dependent caspases revealed similar results. Treatment with rVP1 decreased the expression of pro-caspase-9 and increased the cleavage of both caspase-3 and -7 in BNL cells, whereas no change in the expression of pro-caspases-9, -3, and -7 was observed in AML12 cells within 4 hours of 1 µM rVP1 treatment. Collectively, these results corroborate the data obtained in the MTT assay and flow cytometry (Figures 1A and B).

### rVP1-induced apoptosis is associated with diminished Ku70-Bax interaction

**Figure 2:**
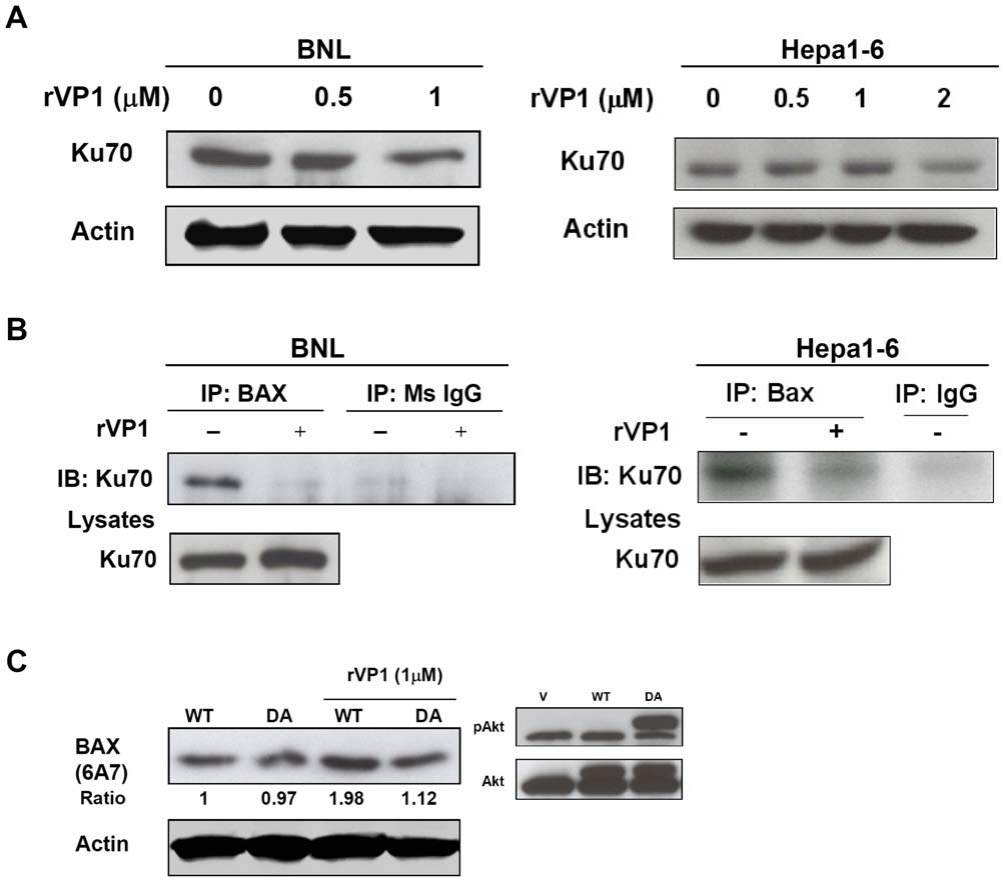
Bax is activated and dissociates from Ku70 in response to rVP1 treatment. (A) Akt inhibits rVP1-induced conformational change of Bax. BNL cells were transfected with either wild-type (WT) or dominant active Akt (DA) and cultured for 24 h. Cells were then treated with or without 1 µM rVP1 diluted in serum-free medium for 1 h and analyzed by western blotting using 6A7 monoclonal antibody (detecting conformationally active Bax). (B) The level of Ku70 decreases after rVP1 treatment. BNL and Hepa1-6 cells were treated with rVP1 diluted in medium containing 1% FBS, as indicated, for 24 h. (C) rVP1 treatment diminishes the interaction between Ku70 and Bax. BNL cells were treated with or without 1 µM rVP1 in serum-free medium for 1 h and Hepa1-6 cells were treated with 2 µM rVP1 diluted in medium containing 1% FBS for 4 h. Cell lysates were immunoprecipitated with anti-Bax antibody and immunoblots were probed with anti-Ku70 antibody.

Earlier studies have shown that Akt inhibits Bax-mediated apoptosis by maintaining the binding of Bax to Ku70 in the cytosol and thus preventing its translocation to mitochondria [15]. Furthermore, a decrease in the level of Ku70 in response to apoptotic stress can release Bax from inhibition [14]. As detailed above, rVP1 treatment reduced Akt phosphorylation and increased the cleavage of mitochondria-dependent caspases. We thus became interested in the effect of rVP1 on the interaction between Bax and Ku70. We first examined the expression of Ku70 in rVP1-treated BNL and Hepa1-6 cells. As shown in Figure 2A, rVP1 treatment decreased the level of Ku70 in both HCC cell lines. A decrease in Ku70 expression was also demonstrated in BNL cells treated with a pan-kinase inhibitor, staurosporine (STS), as a positive control (data not shown). The diminished interaction between Ku70 and Bax was further confirmed by immunoprecipitation analysis. Treatment of rVP1 significantly decreased the association between Ku70 and Bax in BNL and Hepa1-6 cells (Figure 2B). Upon induction of apoptosis, Bax undergoes a conformational change and becomes an activated form [15], which can be recognized by antibody 6A7. By using this antibody, we also demonstrated that rVP1 treatment, which diminished Ku70-Bax interaction, increased the expression of active Bax and active Akt inhibited this activation, as shown in BNL cells transiently transfected with a dominant active Akt plasmid (Figure 2C). These data provide evidence that rVP1-induced Bax activation is Akt-dependent.

### Intratumoral therapy with rVP1 suppresses tumor growth and induces apoptosis of HCC BNL allografts in BALB/c mice

**Figure 3:**
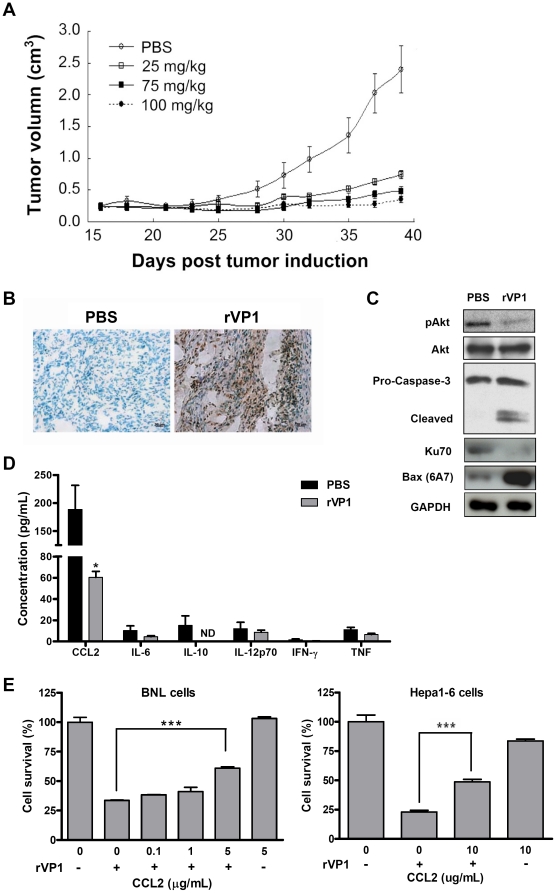
Intratumoral injection of rVP1 attenuates subcutaneous BNL tumor growth by inducing apoptosis and decreasing the level of CCL2. (A) Dose-dependent effect of rVP1 on tumor growth. BALB/c mice bearing subcutaneous BNL tumors with a mean volume of 0.25 cm^3^ received 25, 75, or 100 mg/kg doses of rVP1 intratumorally three times a week for 3 weeks. Mice injected with PBS served as controls. Tumor volume was measured 3 times per week at the time points indicated. Tumor growth was significantly inhibited in rVP1-treated groups (*P*≤0.001 for 75 and 100 mg/kg dose groups, and *P*<0.05 for the 25 mg/kg dose group on day 39) as compared to the PBS group (n = 5). Data shown are representative of three independent experiments. (B) Immunohistochemical analysis of apoptosis in BNL tumors. Apoptosis was detected by TUNEL assay. Immunoreactivity was detected using DAB substrate, which produces an intense brown stain. Negatively stained cells show a greenish-blue color from the counterstain. Images were taken at 400× magnification and are representative samples from rVP1-treated (75 mg/kg) and control (PBS) mice sacrificed one day after the final rVP1 treatment. Specimens were also collected for analyses shown in (C). Scale bars: 20 µm. (C) Western blot analysis of subcutaneous BNL allografts. Total lysates were obtained from tumor specimens of BALB/c mice (75 mg/kg of rVP1). Data shown are representative of three independent experiments. (D) Serum levels of CCL2, IL-6, IL-10, IL-12, IFN-γ, and TNF in rVP-treated (75 mg/kg) and control mice. Blood samples were collected after the completion of the full course of rVP1 treatment. ND: not detected, *P<0.05. (E) Recombinant mouse CCL2 protein (rmCCL2) reverses the inhibitory effect of rVP1 on HCC cell growth. BNL and Hepa1-6 cells were treated with 0.5 and 1 µM of rVP1 in serum-free DMEM in the absence/presence of rmCCL2 for 16 and 24 h, respectively, and assayed for viability by MTT.

To further test the effects of rVP1 on HCC cells *in vivo*, HCC was established by subcutaneous implantation of BNL cells into BALB/c mice. Tumors of approximately 250 mm^3^ were detected two weeks after injection of BNL cells. Four groups of mice were then injected intratumorally with various doses of rVP1 (25 mg/kg, 75 mg/kg, or 100 mg/kg) or PBS three times a week for three weeks. Untreated mice (those receiving PBS) reached the maximum tumor size allowed under the animal care guidelines (approximately 2.5 cm^3^) 39 days after tumor induction (Figure 3A); therefore, tumors of all groups were compared at that time point. Mice treated with rVP1 at doses of 25 mg/kg had significantly smaller tumors than control mice, with higher doses of rVP1 resulting in even smaller tumors. A few mice treated with high doses of rVP1 (75 mg/kg and 100 mg/kg) showed complete tumor regression with no measurable tumor mass.

We further examined apoptosis in the BNL tumor implants by immunohistochemistry and western blot analyses. Tumor specimens collected from mice sacrificed at the end of rVP1 treatment revealed that intratumoral injection of rVP1 substantially increased the number of apoptotic tumor cells as demonstrated by terminal deoxynucleotidyl transferase dUTP nick end labeling (TUNEL) assay (Figure 3B). A decrease in Akt phosphorylation and Ku70 expression as well as an increase in the expression of active Bax and cleavage of caspase-3 were observed in rVP1-treated BNL subcutaneous implants (Figure 3C). These data suggest that increased apoptosis directly contributed to the reduced tumor growth observed in rVP1-treated mice. In addition, we measured the serum levels of various chemokines/cytokines, i.e., CCL2/MCP-1, IL-6, IL-10, IL-12, IFN-γ, and TNF in mice treated with or without rVP1 using cytometric bead array assay. The results showed that mice treated with rVP1 had lower concentrations of CCL2 in sera as compared to the control mice (Figure 3D). In contrast to CCL2, the serum concentrations of the other cytokines tested were relatively low in both control and treated mice. A few of the control mice had a measurable amount of IL-10; however, IL-10 was not detected in any of the rVP1-treated mice. The serum levels of IL-6, IL-12, IFN-γ, and TNF did not vary between treatment groups.

CCL2/MCP-1 (monocyte chemotactic protein-1) is a CC chemokine known to attract macrophages. Recent studies have suggested a role for CCL2 in cell protection [27], [28]. To determine whether CCL2 plays a role in the survival of HCC cells, we examined the effect of recombinant CCL2 protein on the viability of rVP1-treated BNL and Hepa1-6 cells *in vitro*. Figure 3E shows that recombinant CCL2 proteins attenuated the inhibitory effect of rVP1 on cell growth, indicating that the reduced level of CCL2 in rVP1-treated mice may also be involved in the mechanism of delayed tumor progression.

### A decrease in CCL2 production induced by rVP1 mediates cell migration/invasion

**Figure 4:**
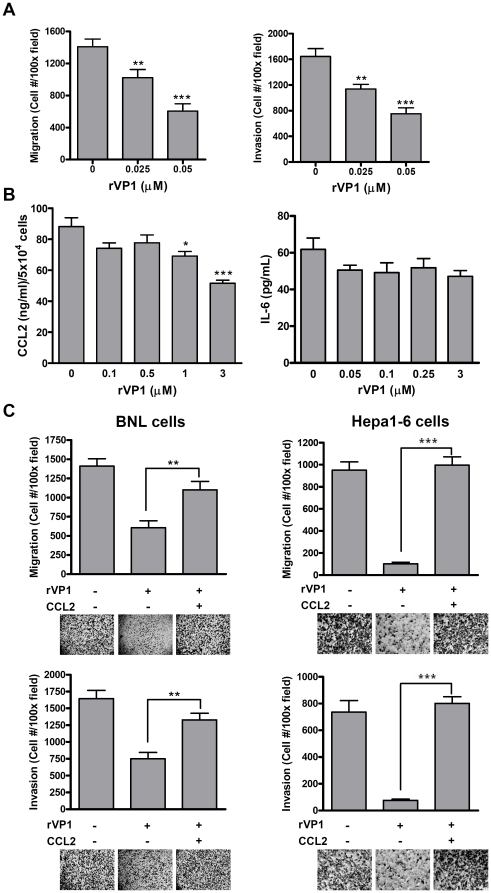
The inhibitory effect of rVP1 on cell migration and invasion is mediated by CCL2. (A) rVP1 inhibits BNL cell migration and invasion in a dose-dependent manner as measured by Boyden chamber assay. (B) rVP1 dose-dependently reduces CCL2 but not IL-6 production by BNL cells. Cells were treated with rVP1 diluted in DMEM with 10% FBS as indicated. After incubation for 48 h, supernatants were collected and the level of CCL2 was measured by ELISA. Values were normalized to viable-cell numbers determined by MTT assay. (C) RmCCL2 attenuates the anti-metastatic and anti-invasive effect of rVP1. BNL and Hepa1-6 cells were treated with or without 0.05 µM rVP1 and 5 µg/mL rmCCL2 for 24 h.

Inhibition of metastasis is still a focus of research on cancer therapy. Given that cancer cell migration and invasion are crucial for metastasis, we examined whether rVP1 had any effect on BNL migration and invasion. As shown in Figure 4A, rVP1 used at a concentration as low as 0.025 µM inhibited both cell migration and invasion. This concentration is 40 times lower than that used in the apoptosis experiments described above (1 µM) and did not affect the viability of normal AML12 hepatocytes (Figure 1A). Our data so far indicate that when used at 1 µM, rVP1 induced apoptosis in BNL cells *in vitro*, whereas a much lower concentration of rVP1 (0.025 µM) exhibited inhibitory effects on cell migration and invasion. Both CCL2 and IL-6 have been implicated in the process of cancer metastasis [25], [36]. In the subcutaneous HCC model, a decrease in serum level of CCL2 was observed in rVP1-treated mice (Figure 3D). To examine the role of CCL2 and IL-6 in rVP1-mediated inhibition of cell migration and invasion, we first measured their levels in culture supernatants of BNL cells by ELISA. A high level of CCL2 was detected in the supernatants, and rVP1 treatment reduced CCL2 secretion by BNL cells in a dose-dependent manner (Figure 4B). However, IL-6 production was not affected by rVP1 treatment. When BNL and Hepa1-6 cells were treated with rVP1 in the presence of recombinant CCL2 protein, the inhibitory effects of rVP1 on cell migration and invasion were reversed (Figure 4C), suggesting that CCL2 took part in the migration/invasion processes of these cells.

### Dephosphorylation of IKK and impairment of NF-κB activation by rVP1 contributes to CCL2 reduction

**Figure 5:**
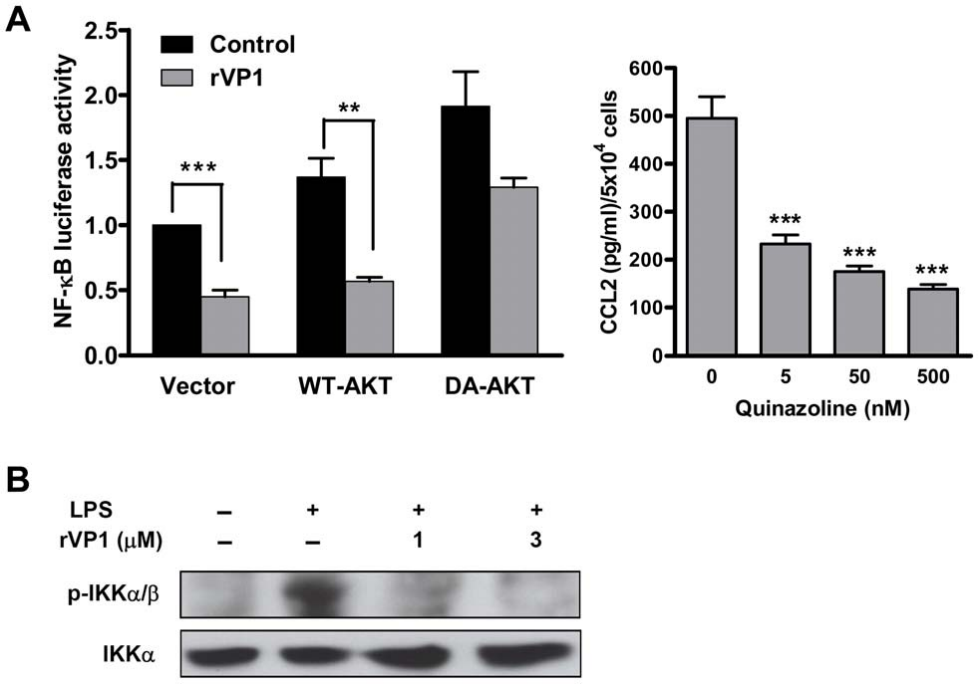
Treatment with rVP1 decreases NF-κB activation that modulates CCL2 production. (A) Left panel: rVP1 inhibits NF-κB activation in an Akt-dependent manner. BNL cells transfected with empty vector, wild-type Akt (WT-AKT) or dominant active Akt (DA-AKT) were co-transfected with a NF-κB-luc reporter plasmid and a vector carrying the EGFP gene for 24 h. Cells were then treated with rVP1 diluted in medium containing 0.5% FBS for 6 h and lysed for NF-κB luciferase assay. The determined values of luciferase activity were normalized to EGFP expression. Each treatment was performed in triplicate, and data shown are representative of at least three independent experiments. Right panel: CCL2 production in BNL cells treated with InSolution™ NF-κB activation inhibitor (quinazoline derivative). The culture supernatants were assayed after incubation for 2 days. (B) Western blots of phospho-IKK. BNL cells were treated with 10 µg/ml LPS in the presence/absence of rVP1 for 30 min.

Since CCL2 has been reported to be regulated by NF-κB in both normal and cancer cells [37], [38], we further examined the effect of rVP1 on NF-κB activation and determined whether it is also mediated by Akt. As shown in Figure 5A, treatment of rVP1 decreased NF-κB activity in BNL cells, which was reversed by transfecting cells with a dominant active Akt plasmid. These results indicate that Akt is, at least in part, involved in the signaling that leads to deactivation of NF-κB induced by rVP1. Furthermore, we confirmed that NF-κB directly regulated CCL2 production in BNL cells, as a significant decrease in CCL2 production was observed in cells treated with InSolution™ NF-κB activation inhibitor, a quinazoline derivative (Figure 5A). Hence, we have shown that rVP1 inhibited Akt phosphorylation (Figures 1C and D) and NF-κB activation. To further investigate the signaling between these two molecules, we examined the effect of rVP1 on IKK phosphorylation. In line with the results of Akt phosphorylation and NF-κB activation, IKK phosphorylation was inhibited by rVP1 (Figure 5B), suggesting that decreased NF-κB activation and the subsequent drop of CCL2 production induced by rVP1 was mediated by Akt and its downstream IKK activity.

### Intravenous administration of rVP1 prolongs survival in BNL orthotopically allografted BALB/c mice

**Figure 6:**
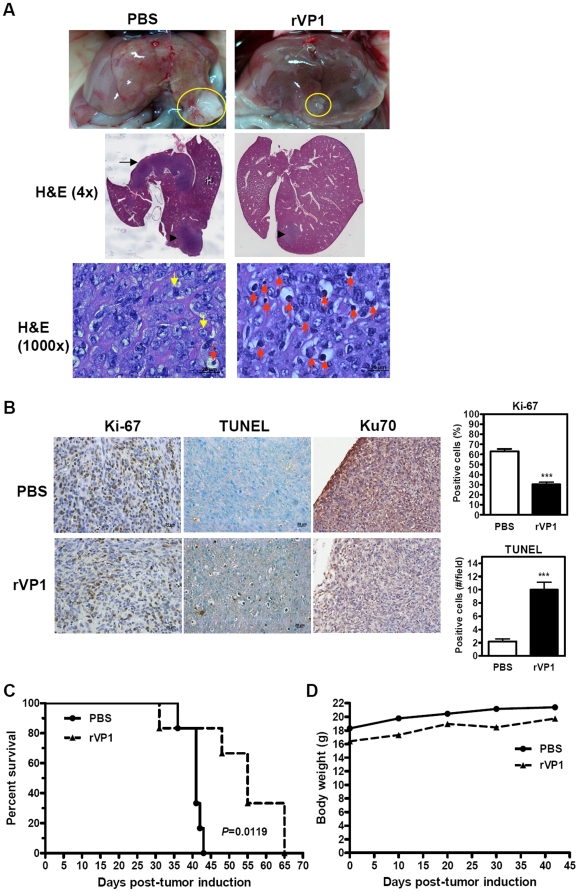
Intravenous injection of rVP1 suppresses tumor progression in an orthotopic allograft mouse model of HCC. (A) Macroscopic photographs and H&E staining of representative orthotopic tumors from two treatment groups. Orthotopic BNL tumor allografts were established in BALB/c mice. Twenty-four hours after surgery mice were treated with rVP1 (25 mg/kg) or PBS via tail vein (n = 6/group). Treatments were performed three times per week until no control mice survived. Specimens are from mice sacrificed after 5 rVP1 treatments and the presence of tumor was confirmed (yellow circles). H&E sections (4×) show the loci of BNL implantation (arrowheads) and intra-hepatic metastasis in a control mouse (arrow). Abundant apoptotic tumor cells characterized by cell shrinkage and chromatin condensation (red arrows) were observed in the rVP1 group at 1000× magnification. The yellow arrows represent mitotic cells. (B) Immunohistochemical analyses of proliferation, apoptosis and Ku70 expression of orthotopic tumors. Tumor proliferation and Ku70 expression were detected with Ki-67 and Ku70 antibodies, respectively, and apoptosis was analyzed by TUNEL assay. Ki-67 positive cells were calculated as number of positive (brown) cells×100/total number of cells counted. TUNEL-positive cells were calculated as number of positive cells (showing brown staining or apoptotic morphology) per field at 400× magnification (scale bars: 20 µm). ^***^ *P*≤0.001 (Student's *t* test). (C) Kaplan-Meier survival curves (*P*<0.05, log-rank test). (D) Mean body weight for each treatment group measured at an approximately 10-day interval.

To investigate the inhibitory effect of rVP1 on metastasis *in vivo*, we established an orthotopic HCC model by implanting BALB/c mouse livers with BNL cells. Twenty-four h after implantation, mice were treated with rVP1 (25 mg/kg) by intravenous injection three times a week until no control mice that received PBS injection survived. Tumor development was examined after five rVP1 treatments. Representative macroscopic images of orthotopic HCC are shown in Figure 6A. We observed a prominent difference in tumor size between the treatment groups (top panel, yellow circles), indicating that intravenous injection of rVP1 inhibited orthotopic tumor growth. Notably, the pathological examination of liver specimens from control mice by H&E staining revealed intra-hepatic metastasis (Figure 6A). In three out of four control mice examined, tumor foci were observed in the lobe adjacent to the lobe where BNL cells were implanted. However, tumor metastasis to a lobe other than the implant lobe was absent in rVP1-treated mice. Under high magnification (1000×), a large number of apoptotic cells were observed in the orthotopic tumors of rVP1-treated mice. Cell proliferation, apoptosis and Ku70 expression in the orthotopic tumors were further examined by immunohistochemistry (Figure 6B). Staining for proliferative tumor cells with Ki-67 antibody and for apoptotic cells by TUNEL assay showed that rVP1 significantly decreased the number of proliferative tumor cells and increased the number of apoptotic cells. A decrease in Ku70 expression was also observed in rVP1-treated tumors. Furthermore, mice treated with rVP1 survived longer, and the difference between groups was statistically significant (Figure 6C). Although mice receiving rVP1 injection were initially slightly lighter than control mice as a result of randomized grouping, the body weight of the treated mice did not appear to be affected by administration of rVP1 at a dose of 25 mg/kg when compared to that of control mice during the period of treatment (Figure 6D). Together, these results indicate that rVP1 suppressed the growth of orthotopic BNL tumors, inhibited intra-hepatic metastasis, and increased the survival rate of mice by hindering tumor proliferation and promoting tumor apoptosis.

## Discussion

**Figure 7:**
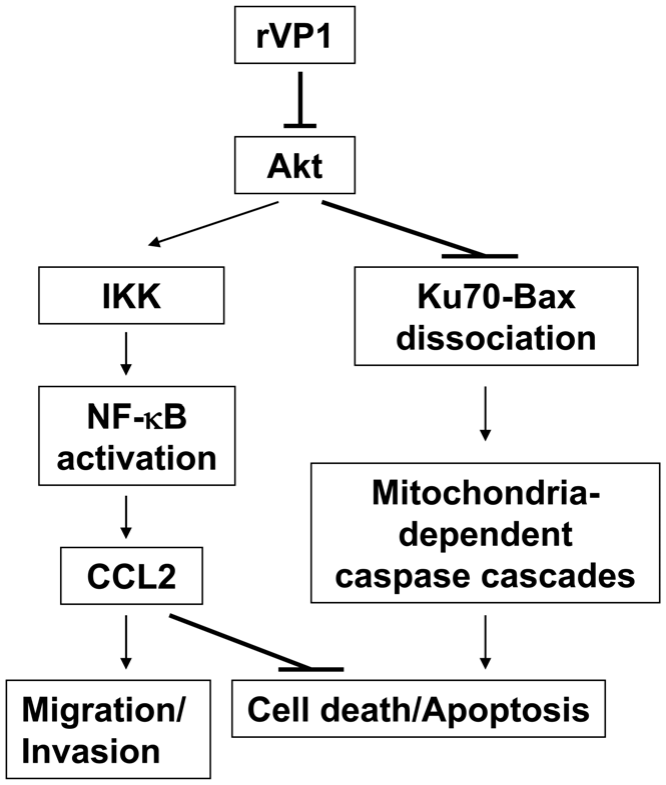
Proposed mechanism of suppression of HCC progression by rVP1. rVP1 inhibits Akt phosphorylation and, through dissociation of the Ku70-Bax complex, activates Bax to promote mitochondria-dependent caspase cascades, leading to apoptosis. The effect of rVP1 on Akt also causes suppression on NF-κB activation and downstream CCL2 production, which enhances cell death and inhibits cell migration and invasion.

Due to the highly chemo-resistant nature of advanced HCC and the consequent limited effective treatment options, the continued development of new therapeutic strategies for HCC is necessary. The exploration of bacterial and viral elements is a direction that should not be overlooked in the search for cancer therapies. Such elements have been shown to inhibit cancer cell growth in human breast cancer MCF-7 cells and mouse melanoma B16-F10 cells [8], [9]. The finding that viral vectors expressing apoptin protein induced significant tumor regression in murine HCC models [9] suggested that further investigation of other viral elements may be warranted for HCC therapy. Here, we show that rVP1 protein of FMDV suppresses HCC progression by modulating two pathways. First, rVP1 induces mitochondria-mediated and caspase-dependent apoptosis via inhibiting Akt phosphorylation and disrupting the association between Ku70 and Bax. Second, by inhibiting Akt phosphorylation, rVP1 also attenuates IKK phosphorylation, NF-κB activation, and CCL2 production, thus suppressing tumor migration/invasion and promoting tumor cell death (Figure 7). The inhibitory effect of rVP1 on tumor growth and intra-hepatic metastasis was further demonstrated *in vivo* using both subcutaneous and orthotopic mouse models of HCC (Figures 3 and 6). To our knowledge, this is the first report of the application of a recombinant viral protein in HCC therapy.

Toxicity to normal tissues limits current cancer therapies, and agents with tumor-specific actions are much desired. Some studies have demonstrated that the adenovirus-derived protein E4orf4 and the chicken anemia virus-derived protein apoptin induce apoptosis in tumor/transformed cell lines, but not in normal cells [10], [11]. Although high concentrations of rVP1 caused AML12 cell death (Figure 1A), we found here that the majority of AML12 cells were still viable when rVP1 was used at a concentration that killed all BNL cells. This differential effect suggests that there may be a dose of rVP1 that would be therapeutically effective against cancer cells, while still maintaining a very low toxicity towards normal hepatocytes. Further investigation needs to be conducted to verify this assumption.

The induction of apoptosis in tumor cells is a major therapeutic strategy for cancer. Flow cytometric analysis of annexin V-PI co-labeling demonstrated that rVP1-induced apoptosis of HCC cells (Figure 1B). Immunohistochemical analysis of rVP1-treated subcutaneous tumors revealed intense apoptosis (Figure 3B), suggesting that the inhibitory effect of rVP1 on tumor growth was mainly due to apoptosis of tumor cells. To further investigate the mechanisms underlying this effect, we first examined Akt phosphorylation and caspase cleavage in response to rVP1 treatment. The association between PI3K/Akt pathway activation and malignant transformation and anti-apoptotic signaling is well known [39], [40]. Activation of the Akt/mTOR signaling pathway occurs in approximately half of the patients with HCC [7]. Although we did not test if mTOR is also a target of rVP1, our results so far suggest that, in BNL and Hepa1-6 cells, rVP1 treatment downregulates Akt and induces apoptosis via the caspase pathway. We observed a clear difference in the effect of rVP1 on cleavage of pro-caspases-9, -3 and -7 in BNL and AML12 cells. In contrast to BNL cells, cleavage of these pro-caspases did not occur in AML12 cells within 4 h of treatment with 1 µM rVP1. Future investigations aimed at elucidating the distinct responses of HCC and normal hepatocyte cell lines to rVP1 treatment may provide the possibility of uncovering a therapeutic target that can minimize the detrimental effect of cancer therapy on normal hepatocytes.

Bax belongs to the Bcl-2 gene family which includes at least 20 pro- and anti-apoptotic genes [41]. Overexpression of the anti-apoptotic gene *bcl-X_L_* and downregulation of *bax* and *bcl-X_s_*, two pro-apoptotic members of the family, have been reported in HCC [42], [43]. In this study, we found that rVP1 promoted Bax activation that stemmed from the dissociation of Bax from Ku70 and was associated with a decrease in the level of Ku70. Our results thus support previous findings suggesting that downregulation of Ku70 is beneficial for cancer therapy [44], [45]. Overall, the findings shown in Figures 1 and 2 indicate that rVP1 treatment inhibits Akt phosphorylation, which causes dissociation of the Ku70-Bax complex. The dissociation of this complex and conformational changes of Bax promote the translocation of Bax to the mitochondria. This process in turn triggers activation of caspases-9, -3, and -7, which leads to apoptotic cell death. The effect of rVP1 on Bax and Ku70 may thus aid in shifting the dysregulated balance of apoptosis/survival signals in HCC.

Recently, much attention has been focused on molecular targeted therapies for HCC. Sorafenib has shown survival benefits in patients with advanced HCC in phase III clinical trials and thus represents the first systemic therapy to be found effective in advanced HCC cases. Although sorafenib provided survival benefit, a corresponding impact in tumor shrinkage has not been observed [46]. To evaluate the potency of rVP1 on tumor shrinkage, we first established a subcutaneous HCC model and administered rVP1 by intratumoral injection. Our results showed not only a dose-dependent inhibition of tumor growth, but also complete tumor regression in some mice treated with high doses of rVP1. In an orthotopic model, intravenous injection of rVP1 prevented growth of orthotopic tumors, inhibited intra-hepatic metastasis and increased survival rate. In contrast to the large number of apoptotic cells observed in the orthotopic tumors of rVP1-treated mice (Figure 6A), normal liver tissue of these mice did not reveal any significant apoptosis (Figure S1). This is somehow different from the *in vitro* results which show that rVP1 treatment causes cell death in some AML12 cells (Figures 1A and B). A likely explanation is that normal hepatocytes, in the presence of surrounding microenvironment, are more resistant to rVP1 *in vivo*. As both HCC models were established in BALB/c mice, these data suggest that under “immune-competent conditions” rVP1 is able to delay tumor progression and tumor size in a manner that affects mouse survival. We speculate that, in addition, the anti-metastatic effect of rVP1 seen in the orthotopic model may play an important role in determining survival outcomes. Since intra-hepatic metastases are a major hallmark of metastatic HCC contributing to dismal long-term survival in HCC patients [47], [48], how rVP1 interferes with the metastasis of HCC and its potential application in anti-metastasis therapy is worth further investigation.

Research interest in developing cancer therapeutics that target CCL2 is increasing rapidly. In the present study, we showed that rVP1 treatment decreased the serum level of CCL2 in a subcutaneous murine HCC model. Our data also revealed that recombinant CCL2 proteins inhibited the anti-growth activity and anti-metastatic as well as anti-invasive effects of rVP1. Based on these findings, we suggest that the decrease in CCL2 production by BNL cells after rVP1 treatment is at least in part associated with the antitumor activity of rVP1. We propose that CCL2 may affect two aspects of tumor development by enhancing the pro-apoptotic function of rVP1 and by contributing to the absence of intra-hepatic metastasis (between lobes) in rVP1-treated mice. Since CCL2 can attract immunosuppressive Tregs and myeloid-derived suppressor cells to the tumor microenvironment [49], treatment with rVP1 may also help overcome the evasion of anti-tumor immune response.

NF-κB is a transcription factor downstream of Akt that can be activated by a variety of stimuli such as TNFα, IL-1, LPS, and viral proteins [50], [51]. It is also involved in the embryonic development of the liver and during liver regeneration [52]. It has been demonstrated that the NF-κB pathway is constitutively activated in HCC tissues [53]. Figure 5 shows that rVP1 treatment impairs NF-κB activation in an Akt-dependent manner. Inhibition of NF-κB has been shown to improve chemotherapy through an increase in apoptosis [54]. The pro-apoptotic activity of rVP1 may render it a suitable candidate for use in combination with other anticancer drugs to offer greater efficacy and less toxicity.

In summary, our study demonstrated that rVP1 suppresses HCC growth, inhibits intra-hepatic metastasis, and prolongs survival in immune-competent mice by inducing apoptosis through inhibition of Akt phosphorylation, dissociation of Ku70-Bax, and activation of caspases-9, -3, and -7. The decrease in CCL2 production induced by rVP1 is also involved in the suppression process. These results suggest that rVP1 has the potential to be developed as a novel therapeutic agent for HCC and support further evaluation of its antitumor properties.

## Supporting Information

Figure S1 **H&E staining of normal liver tissue from mice orthotopically implanted with BNL cells.** In both PBS- and rVP1-treated mice, no significant apoptosis was observed in normal liver tissue after 5 intravenous injections of rVP1 (25 mg/kg). Images of three individual mice from each group were taken from the lobe where BNL cells were implanted (200× magnification).
(TIF)
