# Supplementary figures and images for: Recombinant VP1, an Akt Inhibitor, Suppresses Progression of Hepatocellular Carcinoma by Inducing Apoptosis and Modulation of CCL2 Production
Source: PLoS One. 2011 Aug 3;6(8):e23317. doi: 10.1371/journal.pone.0023317 (PMC3149645; doi:10.1371/journal.pone.0023317)

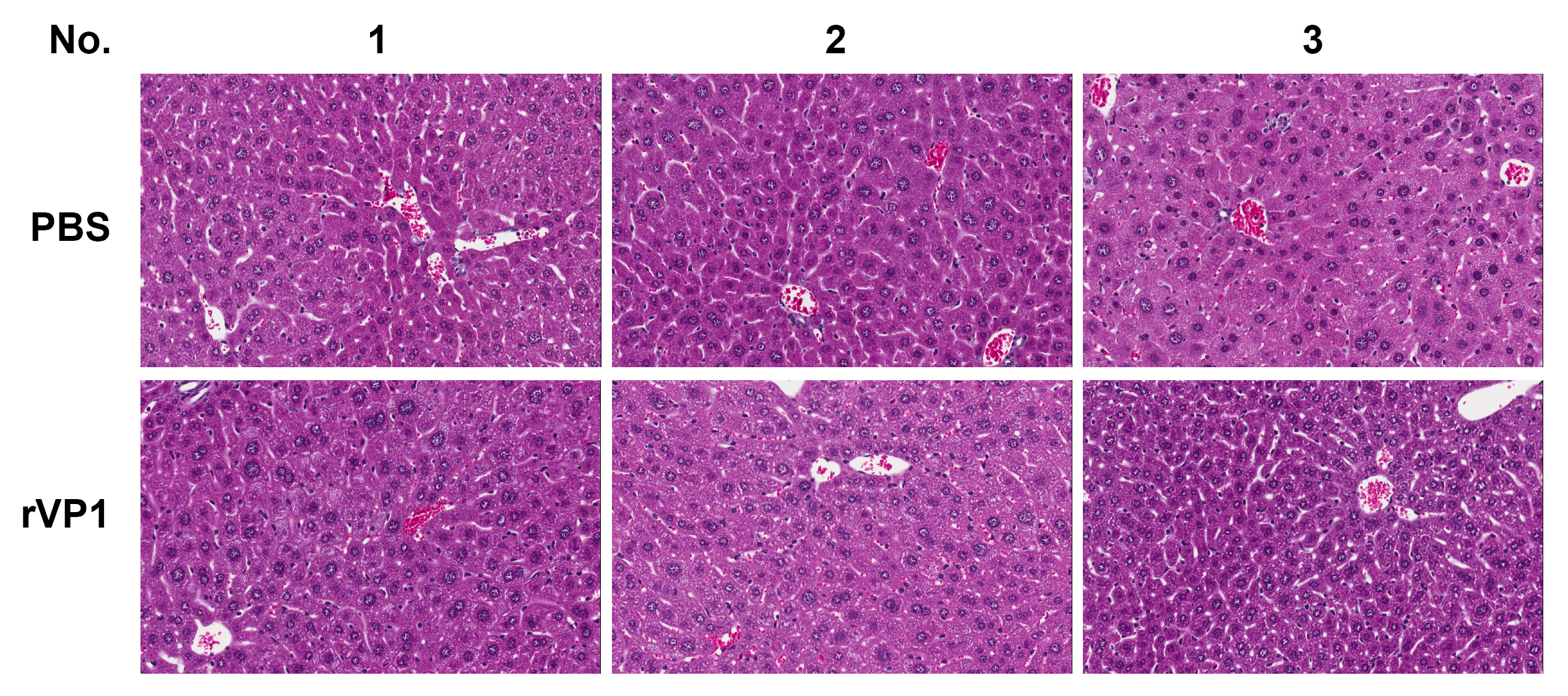

Supplement: Figure S1 — H&E staining of normal liver tissue from mice orthotopically implanted with BNL cells. In both PBS- and rVP1-treated mice, no significant apoptosis was observed in normal liver tissue after 5 intravenous injections of rVP1 (25 mg/kg). Images of three individual mice from each group were taken from the lobe where BNL cells were implanted (200× magnification). (TIF) [file pone.0023317.s001.tif]
